# Selectivity and Plasticity in a Sound-Evoked Male-Male Interaction in *Drosophila*

**DOI:** 10.1371/journal.pone.0074289

**Published:** 2013-09-24

**Authors:** Jeonghyeon Yoon, Eriko Matsuo, Daichi Yamada, Hiroshi Mizuno, Takako Morimoto, Hiroyoshi Miyakawa, Setsuo Kinoshita, Hiroshi Ishimoto, Azusa Kamikouchi

**Affiliations:** 1 Graduate School of Science, Nagoya University, Nagoya, Aichi, Japan; 2 School of Life Sciences, Tokyo University of Pharmacy and Life Sciences, Hachioji, Tokyo, Japan; 3 ProMedico Co., Ltd., Shinjuku, Tokyo, Japan; 4 PRESTO, JST, Chiyoda, Tokyo, Japan; Max-Planck-Institut für Neurobiologie, Germany

## Abstract

During courtship, many animals, including insects, birds, fish, and mammals, utilize acoustic signals to transmit information about species identity. Although auditory communication is crucial across phyla, the neuronal and physiologic processes are poorly understood. Sound-evoked chaining behavior, a display of homosexual courtship behavior in *Drosophila* males, has long been used as an excellent model for analyzing auditory behavior responses, outcomes of acoustic perception and higher-order brain functions. Here we developed a new method, termed ChaIN (Chain Index Numerator), in which we use a computer-based auto detection system for chaining behavior. The ChaIN system can systematically detect the chaining behavior induced by a series of modified courtship song playbacks. Two evolutionarily related *Drosophila* species, *Drosophila melanogaster* and *Drosophila simulans,* exhibited dramatic selective increases in chaining behavior when exposed to specific auditory cues, suggesting that auditory discrimination processes are involved in the acceleration of chaining behavior. Prolonged monotonous pulse sounds containing courtship song components also induced high intense chaining behavior. Interestingly, the chaining behavior was gradually suppressed over time when song playback continued. This behavioral change is likely to be a plastic behavior and not a simple sensory adaptation or fatigue, because the suppression was released by applying a different pulse pattern. This behavioral plasticity is not a form of habituation because different modality stimuli did not recover the behavioral suppression. Intriguingly, this plastic behavior partially depended on the cAMP signaling pathway controlled by the *rutabaga* adenylyl cyclase gene that is important for learning and memory. Taken together, this study demonstrates the selectivity and behavioral kinetics of the sound-induced interacting behavior of *Drosophila* males, and provides a basis for the systematic analysis of genes and neural circuits underlying complex acoustic behavior.

## Introduction

Males of many *Drosophila* species produce a courtship song to attract congener females. A courting male extends a unilateral wing and vibrates it to produce a stereotyped courtship song composed of pulse (i.e., an intermittent series of pulses) and sine (i.e., sinusoidal continuous hums) components toward a target female [1]. Throughout the genus *Drosophila*, these songs are species-specific in their pattern and harmonic content [2], [3]. The songs of *D. melanogaster* comprise a short sine component followed by bursts of pulse components [1], [4]. The inter-pulse interval (IPI), defined as the time between pulses, in the courtship pulse component is approximately 35 ms in *D. melanogaster* and oscillates in a sinusoidal pattern with a 55 to 60-s cycle [5], [6]. On the other hand, *D. simulans* males produce a slightly different pulse component, which has a mean length of 50-ms IPIs with a 33-s sinusoidal period [6], [7]. These IPI parameters significantly affect female courtship receptivity [8]. Indeed, playback experiments revealed that an artificial courtship song containing species-specific IPIs facilitates copulation in a species-specific manner [9].

**Figure 1:**
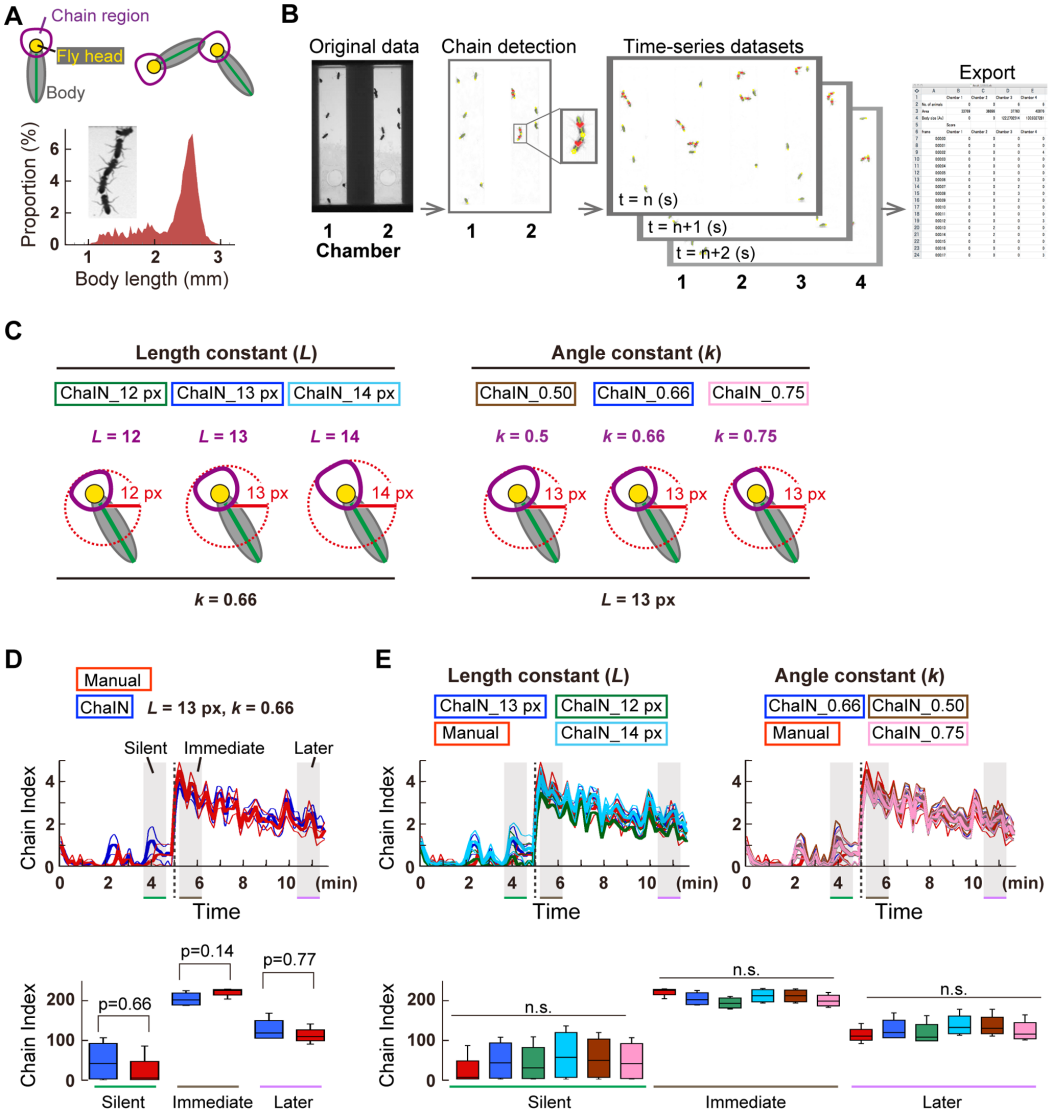
Systematic counting of the chain index by ChaIN. (A) Detection of chaining behavior in fruit flies. In the top panel, green line shows the body axis of each fly. Passive recipients (the leader of the train of flies) are included in the score [11], so that the chain index in the top panel is defined as 2. Bottom panel shows a histogram for the body length of *D. melanogaster* (mean ± standard deviation 2.27 mm ±0.44 mm; n = 17,184 silhouettes of files). Inset image shows the flies forming chains. (B) Flowchart of ChaIN. Chain indices in images were exported as a time-series dataset. (C) Length and angle constants. Change in the length constant (*L*) varies the scale of the chain region (Left panel). Change in the angle constant (*k*) varies the expanse of the chain region (Right panel). Note that ChaIN_13 pixels (px) in the left panel and ChaIN_0.66 in the right panel show the same chain region (*L* = 13, *k* = 0.66). (D) Evaluation of ChaIN. Blue and red traces show the time-course of the chain index counted by ChaIN and an experimenter's manual inspection, respectively (Top panel). Sound playback starts at 5 min (dotted vertical line). Thick and thin lines represent mean ± standard error. Time windows for three temporal phases are hatched in gray. The box plot depicts the cumulative chain indices during three temporal phases of 1 min each (Bottom panel). (E) Change in the length and angle constants had less impact on the chain index. Length constant *L*, originally set as 13 pixels, was shifted to 12 or 14 pixels (Top left panel). Angle constant *k*, originally set as 0.66, was shifted to 0.5 or 0.75 (Top right panel). Box plot shows the cumulative chain indices during three temporal phases (Bottom panel). n. s., not significant.

In a single-sex group situation, female flies gradually decrease their locomotor activity when they are exposed to synthetic full courtship songs or just a component of a courtship song, whereas male flies increase their locomotor activity and also show intensive homosexual courtship activities displayed as chaining behavior [10]. Male behavior is affected by many signal cues derived from female flies, such as visual stimuli, chemical substances, and physical/mechanical interactions when males and females are within proximal distance. The single-sex group assay is useful for analyzing auditory effects on male sexual behavior because it minimizes the female-derived signals. Chaining behavior is the most obvious phenotype of male flies under unisexual conditions upon exposure to the courtship song. The song playback experiment is established to quantify the intensity of the chaining behavior, by counting the number of flies forming male-male courtship chains [11]–[13] (Fig. 1A inset). This auditory response of males is thus a simple and powerful approach to screen genes or neural circuits required for the auditory system in *D. melanogaster* flies. Chaining behavior is typically measured manually using video-recorded data of the fly behavior. The manual measuring method is problematic for systematic analysis of chaining behavior, however, as it requires frame-by-frame counting of the chaining behavior. Considering that visual inspection of chain indices in a movie of a single 12-min experiment requires hours even for an expert, manual analysis is time-consuming to apply to a large-scale assay. Moreover, manual detection of chaining behavior is subjective, which increases the variability of the analysis results. Thus, established criteria and a bias-free experimental design are important for obtaining reliable data.

In the present study, we applied our automated monitoring system for analyzing chaining behavior to better characterize the sound-evoked behavior. With this high-throughput method, we first systematically quantified chaining behavior to detect a selective effect of various types of natural and artificial courtship songs on two *Drosophila* species, *D. melanogaster* and *D. simulans*, which are separated by only 2 to 3 million years of evolution [14]. Moreover, we investigated whether a repetitive presentation of courtship songs leads to a decline in chaining behavior, and if so, whether such behavioral plasticity requires *rutabaga* (*rut*), a gene of the cAMP signal pathway that has a crucial role in most habituation, learning, and memory systems [15], [16].

## Results

### Automatic count of male-male courtship chains

For systematic analysis of male-male courtship chains stimulated by courtship song playback from a loudspeaker, we developed a computer program that detects chaining behavior from a digital video recording and quantifies the chaining behavior by automatically calculating the chain index (Fig. 1A, B). Our system, the Chain Index Numerator (ChaIN), detects the locations of individual fly body centroid and head orientations in each video frame and calculates the chain index by counting the number of animals forming individual male-male chains. Previous studies defined flies orienting toward other flies in a proximal position as forming a chain [11]–[13]. In our ChaIN system, we set a chain region, *P*, of each fly as described by:
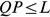
(1)

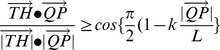
(2)in which *H* is the position of the fly head and *T* is its abdominal tip. *Q* is the rear end of the head area, which was set at a point on line segment *TH*, 5 pixels (0.6 mm) away from *H*. The ChaIN system has two variable parameters, the length (*L*) and angle (*k*) constants, which define the scale and expanse of the chain region, respectively (Fig. 1C). Chain formation was defined as a situation in which the “chain region”, a fan-shaped area surrounding the fly head (depicted as a purple ring in Fig. 1A) overlaps the body of another fly (depicted as a gray ellipse in Fig. 1A). We evaluated the performance of ChaIN by scoring the chain index on a 12-min movie (n = 4 chambers). We first optimized the length and angle constants by assigning them a series of values. When the parameters were set to *L* = 13.0 pixels (1.56 mm) and *k* = 0.66, the ChaIN system produced scores similar to those obtained by an experienced experimenter performing a manual inspection (Fig. 1D). To compare manually derived chain index and that derived by ChaIN during song playback, the time-course of the chain index was divided into three temporal phases (Fig. 1D): the “Silent” phase (basal activity), the “Immediate” phase (stimulation onset), and the “Later” phase (just before the end of stimulation). The cumulative 1-min values of chain indices in these temporal phases revealed a broader distribution of chain index in the ChaIN results (Fig. 1D bottom panel), which can be compensated for by increasing the number of trials. Our ChaIN system simultaneously computes the chain scores in four chambers, and requires only a few minutes to set up and run. On the other hand, an experienced investigator typically requires approximately 4 h to score the chain index manually for four chambers. Our new method is thus reliable and applicable for high throughput analysis. Changes in the constants *L* and *k* did not dramatically affect the chain index (Fig. 1E). We thus used the original values (*L* = 13.0 pixel and *k* = 0.66) for two constants to set the chain region in the following experiments.

**Figure 2:**
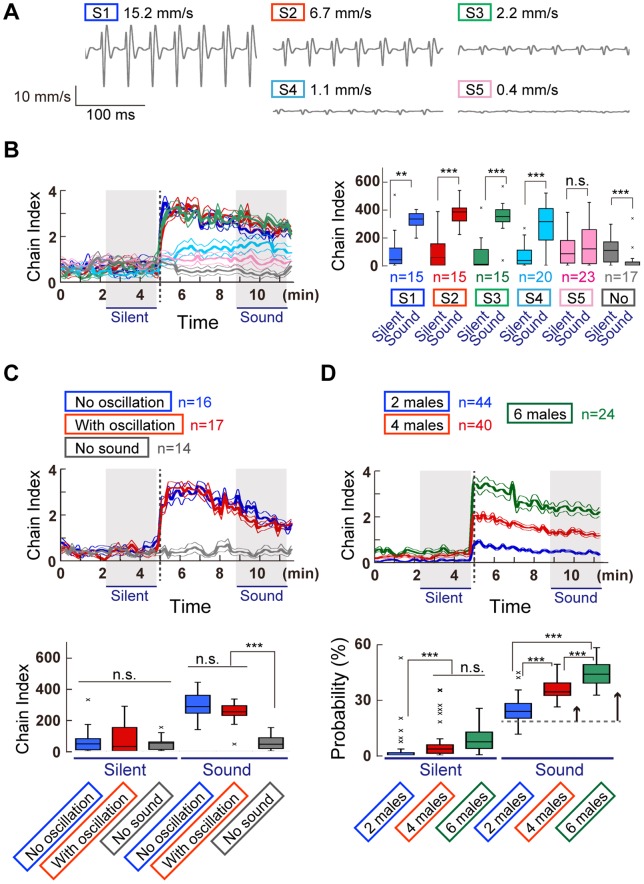
Sensitivity of the chaining behavior. (A) Time traces for the artificial sound playback from a loudspeaker. Mean baseline-to-peak amplitudes of their particle velocities are: 15.2 mm/s in S1 (blue), 6.7 mm/s in S2 (red), 2.2 mm/s in S3 (green), 1.1 mm/s in S4 (light blue), and 0.4 mm/s in S5 (pink). (B) Time-course for the chain index in response to artificial sounds (Left panel; same color-code as Fig. 2A). A baseline response without sound playback is shown as the gray line. Thick and thin lines represent mean ± standard error, respectively. Sound playback begins at 5 min (dotted vertical line). Time windows for silent and sound temporal phases are hatched in gray. Box plot depicts the comparison of chain indices between the silent and sound temporal phases that span 2.5 min each (Right panel). Sound intensity 1.1 mm/s (S4) and greater evokes chaining behavior (Wilcoxon signed-rank test). Basal chaining behavior without sound declined gradually, as shown in the “no sound” group. (C) IPI oscillations have no effect in evoking the chaining behavior. Artificial songs without (blue) or with (red) an IPI oscillation induced significant increases in the chain index (Kruskal-Wallis test followed by Scheffe's multiple comparison) (Bottom panel). (D) Effect of population density on the chaining behavior. Chain indices between two (blue), four (red), and six (green) males during song playbacks are shown as time-courses (Top panel). Box plot shows the mean probability of a fly forming a chain in each second during the “Silent” and “Sound” phases (Kruskal-Wallis test followed by Scheffe's multiple comparison) (Bottom panel). Arrows indicate the increase in the probability from 2 males (24%) to 4 males (36%) or to 6 males (44%). *p<0.05; **p<0.01; ***p<0.001; n. s., not significant. n, number of behavioral chambers examined.

We applied the ChaIN system to analyze the chaining behavior of wing-clipped *D. melanogaster* males exposed to an artificial pulse song containing species-specific sound parameters of this species, i.e., 35-ms IPIs with 167-Hz intrapulse frequency [17], [18] (Fig. 2A). Consistent with a previous study that monitored the male-male courtship evoked by artificial courtship song [10], the chain index in response to various song intensities ranging from 0.4 mm/s to 15.2 mm/s increased in an intensity-dependent manner and was saturated at intensities as high as 2.2 mm/s (Fig. 2B). As expected, the threshold of this chaining behavior (S4 in Fig. 2B, which corresponds to 1.1 mm/s, baseline-to-peak mean) was much higher than the threshold of auditory neurons (5.7×10^−2^ mm/s) or startle behavior (0.12 mm/s) in response to pure tones [19]. Oscillation of the IPIs did not affect the number of chain formations (Fig. 2C), confirming that a rhythmic component in the IPI is not necessary for evoking the male-male courtship, as shown previously [10]. We thus used a constant IPI with the sound intensity at the saturation level (ca. 15 mm/s) for further experiments using synthetic and recorded songs.

We then examined whether the fly population density affects the probability of chain formation. We compared the chain indices obtained from fly groups containing two, four, or six males during the song playback and found that the chain index increased nonlinearly as the number of flies in the chamber increased (Fig. 2D). To confirm this nonlinearity, the cumulative chain index for 2.5-min silent and sound phases was normalized by dividing it by the number of flies in the chamber. Then, the mean probability that a fly forms a chain was calculated by dividing the normalized cumulative chain index by 150, the maximum value for 2.5-min cumulative chain index per fly. The probability of chaining behavior per fly increased significantly in both the silent and sound phases as the number of flies increased, confirming the population-density dependent nonlinearity of chain formation (Fig. 2D).

### Species-selective sound preference

**Figure 3:**
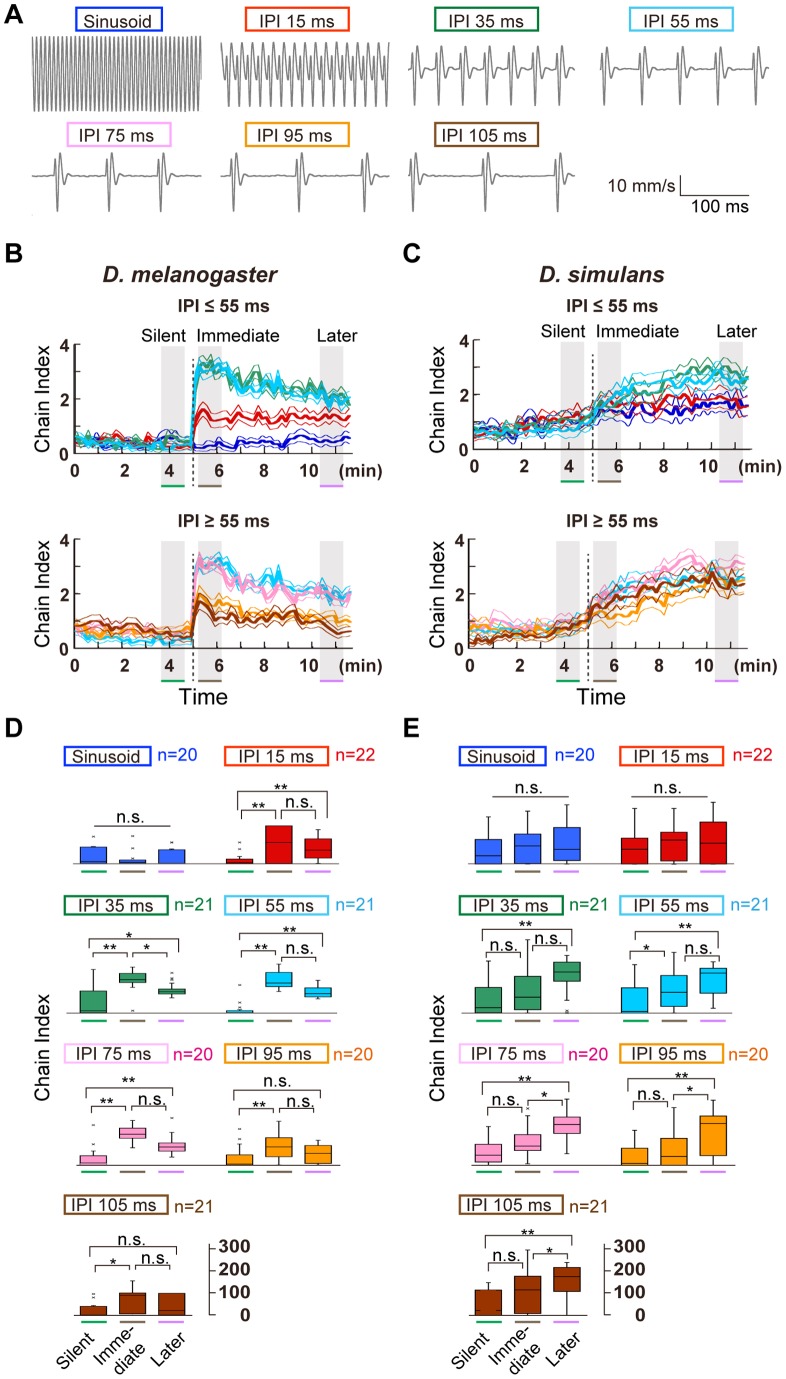
Selectivity to different IPI songs in two *Drosophila* species. (A) Artificial song patterns used in this assay. (B and C) Chain index of *D. melanogaster* (B) and *D. simulans* (C) flies to artificial songs that carry various IPIs. Time-courses to the IPIs shorter (top) and longer (bottom) than 55 ms are shown. Time windows for three temporal phases are hatched in gray. (D and E) *D. melanogaster* (D) and *D. simulans* (E) responses in three temporal phases to artificial pulse songs of varying IPIs. Original time traces are shown in Figure 3B and 3C. Responses to artificial sine song, and pulses of 15, 35, 55, 75, 95, and 105-ms IPIs are color coded in blue, red, green, sky blue, pink, orange, and brown, respectively, in panels (B–E).

Male *D. melanogaster* and *D. simulans* flies generate a species-specific courtship song with a mean IPI of ca. 35 ms and 50 ms, respectively [7]. We applied our ChaIN method to evaluate how species-specific pulse interval parameters, especially IPIs, contribute to chaining behavior using synthetic pulse songs in the playback experiment (Fig. 3A). The time-course of chaining behavior formation differed between these species; whereas *D. melanogaster* males displayed vigorous chaining behavior immediately after the pulse-song playback started, *D. simulans* males began chaining behavior more slowly in response to the sound playback (Fig. 3B, C). To better understand such chaining behavior dynamics during song playback, the cumulative chain indices in the three temporal phases, i.e., silent, immediate, and later phases, were compared. In *D. melanogaster*, characteristic correlations between IPIs and the responsive phase were revealed by comparing the cumulative chain indices between the three phases. IPIs between 15 ms and 75 ms significantly induced chaining behavior at both the immediate and later phases when compared with the index at the silent phase (p<0.05, Friedman's test followed by Scheffe's multiple comparison; Fig. 3D). The longer IPIs (95 and 105 ms) induced chaining behavior specifically at the immediate phase (p<0.05, compared with the silent phase, Friedman's test followed by Scheffe's multiple comparison). Chain index did not increase with sinusoid songs, suggesting that the pulse sound of the courtship song component was required for evoking the male-male courtship chains.

We compared the chain indices between songs with different IPIs in each temporal phase (Fig. S1). In *D. melanogaster*, the difference was larger at the immediate phase than at the later phase; IPIs ranging from 35 ms (typical to *D. melanogaster*) to 75 ms induced significantly higher chain indices than those by the shortest (15 ms) and longest (105 ms) IPIs (p<0.05, Kruskal-Wallis test followed by Scheffe's multiple comparison). Such a difference in the chain indices between IPIs was less prominent at the later phase, probably due to the gradual decrease in the response to preferred IPIs over time (Fig. S1A).

The number of pulses in a single burst (1 s each) differed between different IPI series, i.e., the 15-ms IPI song carries 66 pulses in a burst, whereas the 105-ms IPI song has only 10 pulses in a burst. To exclude the possibility that fewer pulses in a single burst of the105-ms IPI song would be less likely to induce chaining behavior, we increased the pulse number of the 105-ms IPI song to 29 in each single burst, the same as that of the 35-ms IPI stimulus (single burst of 29 pulses with 105-ms IPI lasts 3 s). Comparison of the chain indices between the 10- and 29-pulse 105-ms IPI songs revealed that the number of pulses in a burst had little impact on chain formation (Fig. S2A). This finding confirmed that the IPI rather than the pulse number was the critical parameter for evoking an interaction between males.

On the other hand, *D. simulans* males apparently preferred longer IPIs than *D. melanogaster* (Fig. 3C, E). Unlike *D. melanogaster*, *D. simulans* displayed less chaining behavior during the immediate phase (Fig. 3E). At the later phase, IPIs of 35 ms and longer effectively induced chain formation when compared with the silent phase (p<0.05; Friedman's test followed by Scheffe's multiple comparison). Sinusoid and pulses with a 15-ms IPI did not induce a significant increase in the cumulative chain indices at either the immediate or later phases (p>0.17). The preference for IPIs in *D. simulans* was less prominent than that in *D. melanogaster*, however, as comparisons of the cumulative chain index in each temporal phase revealed no significant differences between songs (p>0.36, Kruskal-Wallis test followed by Scheffe's multiple comparison; Fig. S1B). Experiments with longer exposure to pulse songs with 35, 55, and 75-ms IPIs revealed that *D. simulans* flies decreased their chaining behavior gradually after they reached the maximum chain indices (Fig. S2B). Taken together, the chain formation of flies was induced effectively by specific IPIs, whose range contained species-specific pulse songs. Moreover, *D. simulans* males showed a broader preference for IPIs and slower kinetics than *D. melanogaster* males, manifesting a difference in the auditory behaviors between these species.

**Figure 4:**
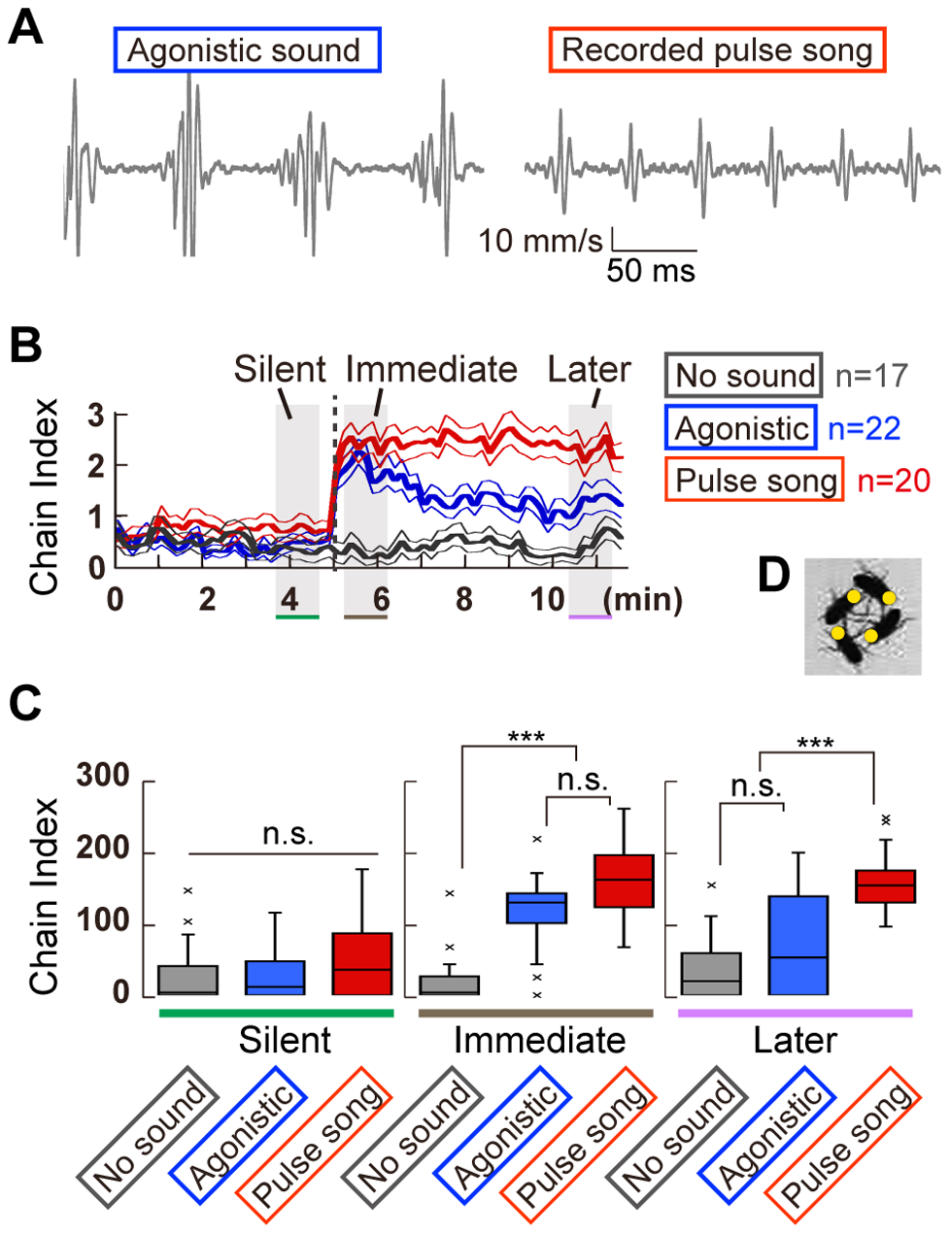
Recorded fly sounds evoke chain formation. (A) Recorded sounds used in this assay. (B) Time-course for the chain index. Responses to an agonistic sound and a pulse song are color-coded in blue and red, respectively. Response without sound (no sound) is shown in gray. (C) The cumulative chain indices during three temporal phases of 1-min, shown in Fig. 4B (Kruskal-Wallis test followed by Scheffe's multiple comparison, ***p<0.005; n. s., not significant). (D) Flies under the agonistic sound. Yellow circles indicate the positions of the fly head.

The pulse sound is the most critical component of the courtship song for inducing chaining behavior. Agonistic behavior is another example of a male-male interaction that produces sound signals in *Drosophila* species by vibrating their wings. In contrast to acoustic courtship signals, agonistic sounds of male *D. melanogaster* comprise exclusively pulses, which carry twice as long and have a more variable IPI than courtship songs [20] (Fig. 4A). Although these two pulse sounds, the courtship song and agonistic sound, share similar sound structures, the effect of the agonistic sound on chaining behavior has not been investigated. To determine if the agonistic pulse sounds affect chaining behavior, we compared chain indices obtained from *D. melanogaster* responses under various sound-playback conditions, such as the recorded agonistic sound, natural courtship pulse songs, and silence during each temporal phase (Fig. 4B). Unexpectedly, agonistic sounds promoted intense chaining behavior at the immediate phase, as observed in natural courtship pulse song condition (p = 0.10 between two songs, Kruskal-Wallis test followed by Scheffe's multiple comparison; Fig. 4C). Whereas under the courtship pulse songs in which flies displayed prolonged high-intensity chain index, the response to the agonistic song rapidly decreased during stimulation (comparison between immediate and later phases yield p>0.2 in the courtship pulse song and p<0.05 in the agonistic song, Friedman's test followed by Scheffe's multiple comparison). The male-male interaction observed under the agonistic-sound playback is likely to be a courtship interaction, because in most cases the head of the chasing fly was oriented to the abdomen of the recipient fly (Fig. 4D). The agonistic song thus evoked an initial induction of the chaining behavior, which rapidly attenuated.

### Behavioral plasticity

**Figure 5:**
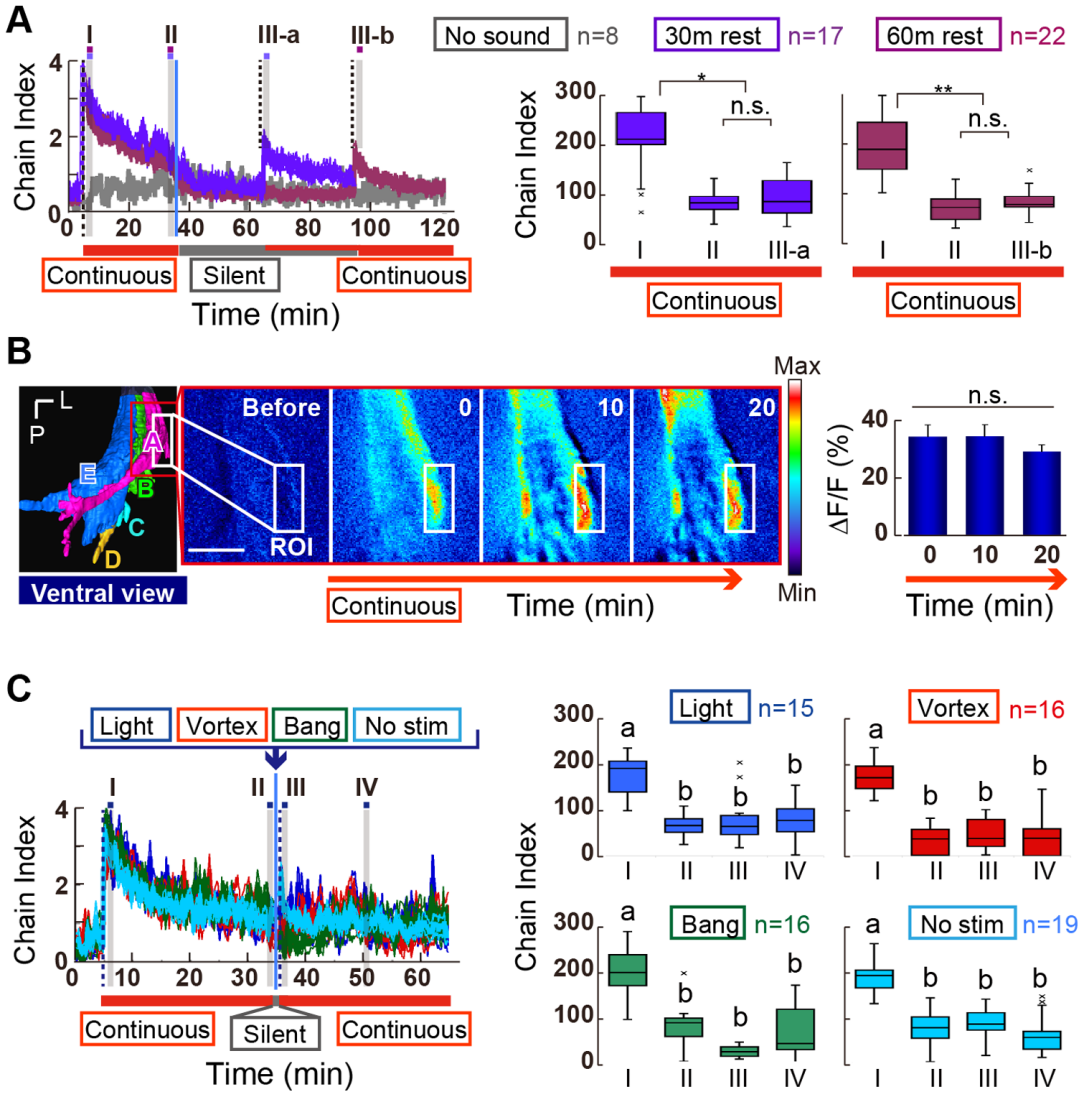
Decrease in the chaining behavior. (A) Retrieval test. Flies exposed to the continuous pulse song for 30 min were maintained in silence for 30 min (bluish-purple) or 60 min (red-purple), and returned to sound-exposure. Dotted vertical lines indicate the start and restart of the sound playback (Left panel). Blue vertical line indicates cessation of the first playback. The chain index for the initial phase (I) was significantly higher than those in later (II) and after the restart (III-a and III-b) temporal phases (Middle and Right panels). (B) GCaMP3 fluorescent change to the continuous pulse song. Left panel shows a ventral view of the AMMC. The AMMC is the primary center for JO neurons and is anatomically subdivided into zones A (magenta), B (light green), C (light blue), D (orange), and E (blue). Pseudocolor images at 0, 10, and 20 min after stimulus onset are shown (middle panels). The region of interest (ROI) was set in the AMMC zone A. 1-min averages of the ΔF/F intensity at 0, 10, and 20 min after stimulus onset was plotted (right panel). No significant difference was observed between them (N = 4, Friedman's test followed by Scheffe's multiple comparison). P, posterior; L, lateral. Scale bar  = 20 µm. (C) Dishabituation tests. Light blink (blue), vortex (red), bang (green), or a short silent period (light blue) does not restore the behavioral suppression to the continuous pulse song (Left panel). A dishabituation stimulus was applied at the start of a 40-s silent interval (arrow). Dotted vertical lines indicate when the sound playback was started (at 5 min) and restarted (at 35 min 40 s). A blue vertical line indicates cessation of the first playback. Right panels show the cumulative chain indices for four temporal phases. **p<0.01; *p<0.05; n. s., not significant.

**Figure 6:**
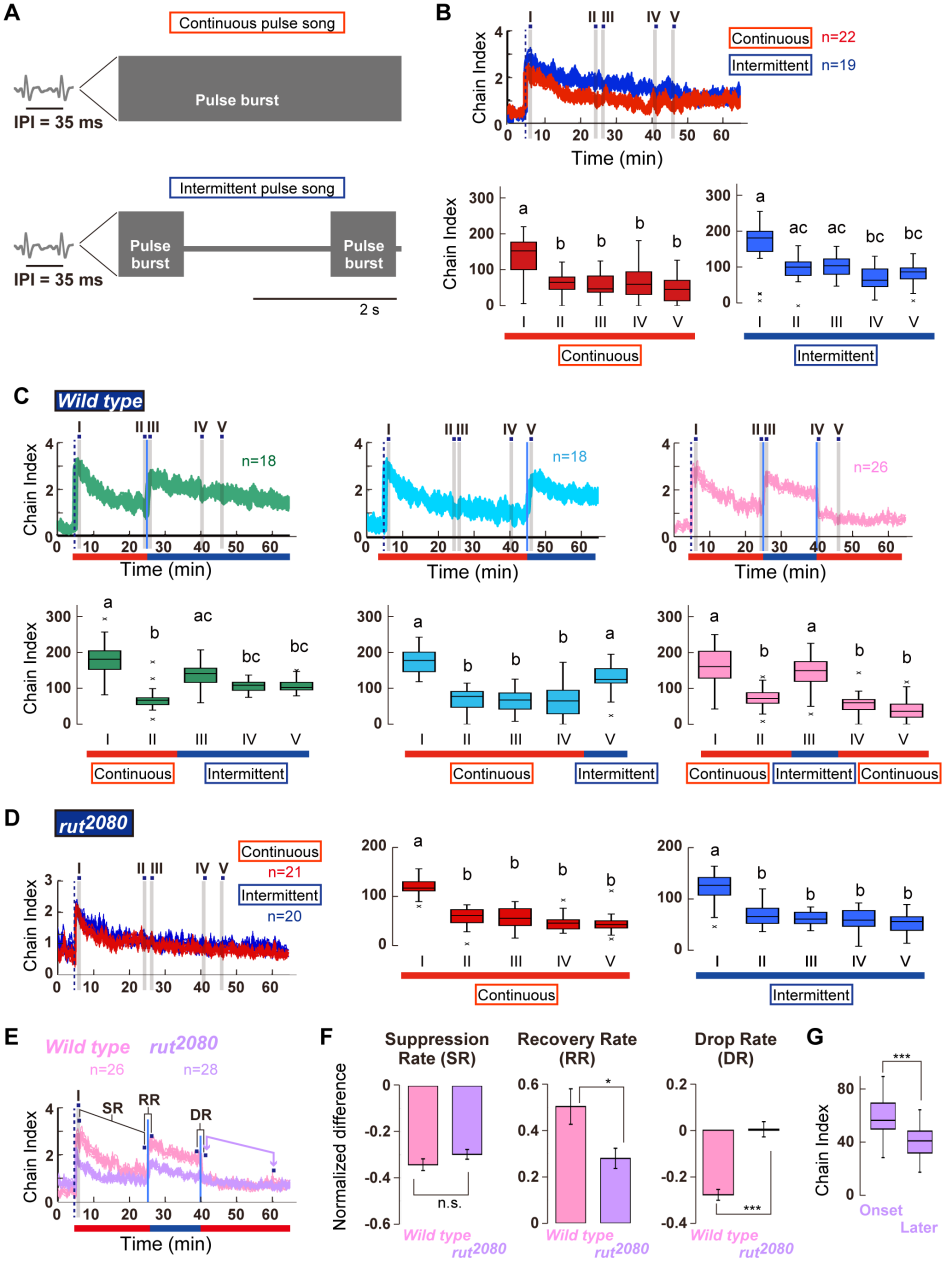
Behavioral change in wild-type and *rut^2080^* mutant flies. (A) Artificial songs used in this assay. (B) Chain index to prolonged pulse sounds. Top panel shows the time-course of the chain index to the continuous pulse song (red line) or intermittent pulse song (blue line). Time windows for counting the cumulative chain index in each 1-min temporal phase are marked with gray boxes (temporal phases I to V). Bottom panels show cumulative chain indices during five temporal phases, marked in the top panel. Responses to the continuous pulse song (Left panel) and intermittent pulse song (Right panel) are color-coded in red and blue, respectively. Different letters indicate significant differences between groups (Friedman's test followed by Scheffe's multiple comparison, p<0.05). (C) Time-course of chain index to acoustic stimuli that contain sequential continuous and intermittent sound clips (Top panels). Time windows of the continuous song (red underline) and intermittent song (blue underline) playbacks are underlined at the bottom of each panel. Blue vertical lines depict the time when the sound clip is switched between two songs. Bottom panels show the box plot of the cumulative chain indices during the five temporal phases. Each temporal phase spans 1 min (indicated in the top panel as temporal phases I to V). (D) Behavior in *rut^2080^* mutant flies. Chain indices to the continuous (red line) and intermittent (blue line) sounds are shown (Left panel). Cumulative chain indices for five temporal phases are shown in the box plot (Middle and Right panels). (E) Chain indices in wild-type and *rut^2080^* mutant flies. SR, suppression rate; RR, recovery rate; DR, drop rate. Magenta arrows indicate two temporal phases for Fig. 6G. (F) Behavioral changes in wild-type and *rut^2080^* mutant flies. *p<0.05; ***p<0.001; n. s., not significant (Student's t-test). (G) Cumulative chain indices for *rut^2080^* mutant flies at the onset and later of the second playback of the continuous pulse song (shown as arrows at 40 min and 60 min in Fig. 6E, respectively).

The artificial courtship song (repeated bursts of species-specific pulse sound) induced intensive chaining behaviors in *D. melanogaster* flies right after playback onset. The chaining behavior, however, gradually decreased over time during song playback (e.g., Fig. 3D). Why does the repeated artificial song induce a decrease in behavior? One possible cause for this is motor/sensory fatigue. To determine whether motor/sensory fatigue occurred during the playback experiment, we allowed the *D. melanogaster* flies a resting period (no sound) after a 30-min playback of a continuous pulse song of 35-ms IPI (Fig. 5A; see Fig. 6A for the stimulus pattern). The chaining behavior gradually decreased to 41% (chain index  = 1.5; Table S1) after a 30-min song playback (Fig. 5A). During the following 30-min resting period, the chaining behavior ceased. When the song playback was restarted, flies again exhibited chaining behavior. The intensity of the resumed chaining behavior was very similar to that observed before the resting period (chain index  = 1.7; Fig. 5A bluish-purple line; Table S1). Similarly, the suppressed chaining behavior did not recover to the original intensity (chain index  = 3.2 at the stimulus onset, chain index  = 1.5 at the stimulus restart; Table S1) even after 60 min of rest (Fig. 5A reddish-purple line). These results suggest that the chaining suppression was not due to motor/sensory fatigue, but likely involved an experience-dependent physiologic change in the neuronal systems.

Sensory adaptation, in which sensory neurons become less sensitive to a repeated stimulus over time, is another possibility. To determine whether the chaining suppression was due to a sensory adaptation of the auditory system, we monitored the calcium response of a primary auditory center, the antennal mechanosenory and motor center (AMMC) zone A, in the brain [21] (Fig. 5B). Time-lapse imaging of the GCaMP3 signal revealed that the calcium signal in response to the receiver's vibration induced by the continuous pulse song was virtually constant for at least 20 min after stimulus onset (p>0.75, comparison between the GCaMP3 signals at 0, 10, and 20 min after stimulus onset, Friedman's test followed by Scheffe's multiple comparison, N = 4; Fig. 5B). Thus, sensory attenuation is not likely the primary reason for the suppressed chaining behavior.

Habituation is defined as a gradual decline in the response to a stimulus following repeated application of the same, or a very similar, stimulus. Habituation, hence, involves diminution of existing responses rather than acquisition of novel responses. To investigate whether the behavioral attenuation was due to habituation, we inserted a 40-s silent interval with a novel stimulus, such as a visual stimulus (blinking light of backlit LED) or a mechanical stimulus (vortexing for 2 s or picking up and banging down the behavioral chamber) as a dishabituation stimulus, between two series of 30-min continuous pulse songs. Such insertions of an irrelevant stimulus did not restore the chaining behavior (Fig. 5C). These results suggest that the chaining suppression to the continuous pulse song was not due to behavioral habituation.

To explore the relationship between the detailed kinetics of the behavioral suppression and the temporal pattern of the stimulus, flies were challenged to a 1-h continuous pulse song or an intermittent pulse song, the latter of which carried constant pulses with a 35-ms IPI and 2-s silent periods between 1-s bursts of pulses (Fig. 6A). The chaining behavior decreased significantly after the first 20-min with exposure to the continuous pulse song, as shown in Fig. 5C (Fig. 6B red line; chain indices at 5 min and 25 min were 2.2 and 1.0, respectively; Table S1). Flies exposed to the intermittent pulse song exhibited a response attenuation with slower rates than flies exposed to the continuous song (Fig. 6B blue line; chain indices at 5 min and 25 min were 2.7 and 1.9, respectively; Table S1). This moderate suppression of chaining behavior in intermittent-song exposed flies possibly correlated with the pulse number that was applied to the flies during the playback experiment. To investigate the effect of pulse number (i.e., stimulus intensity per unit time) on chaining suppression, the flies were exposed to the continuous pulse song with various pulse numbers per second (IPI  = 35, 55, or 75 ms) that were confirmed to induce essentially the same chain index at least for the first 6.5-min song playback (p>0.93, Fig. S1A). As a result, playback of the continuous song with 35, 55, or 75-ms IPIs induced quite similar chaining suppression kinetics up to at least 40-min exposure (Fig. S3), suggesting that the stimulus intensity per unit time was unlikely the major factor in the chaining suppression kinetics.

Intriguingly, the suppression of chaining behavior was immediately released when we altered the temporal pattern of the pulse song during playback; when the song playback was shifted from the continuous pulse song to the intermittent pulse song, chaining suppression recovered to 77% of the chain index immediately after the song playback (Fig. 6C left panel). This response recovery was also observed even after 40-min playback of the continuous song (74% recovery; Fig. 6C middle panel). Furthermore, when the playback song was changed back to the continuous song, the recovered chaining behavior was again severely suppressed (Fig. 6C right panel). In contrast, when the song playback was changed in the opposite manner, i.e., from the intermittent pulse song to the continuous one, the chaining behavior was immediately suppressed (Fig. S4A). These results, together with the result shown in Fig. 5A, confirmed that the chaining suppression was not due to simple fatigue. Finally, the behavioral suppression induced by continuous pulses of 35-ms IPI was not released by a shift of IPI from 35 ms to 55 ms (Fig. S4B). It is possible that in this plastic behavior, flies generalize the pulses of these different IPIs.

Previous studies indicated that *rut*, an adenylyl cyclase gene, affects various neuronal and behavioral plasticities, such as learning and memory associated with environmental cues, including visual, caloric, chemical stimuli, and mechanical stimuli [22]–[26]. We next examined whether *rut* is also involved in the behavioral change induced by continuous fly courtship songs observed in our experimental condition. Flies with a loss-of-function mutant allele of *rut*, *rut^2080^* flies, exhibited chaining behavior in response to the continuous pulse song (Fig. 6D). Although the immediate response of *rut^2080^* flies to the continuous song was smaller than that of wild-type flies, the response of *rut^2080^* flies was gradually suppressed with similar kinetics to that of wild-type flies (Fig. 6D). Unlike wild-type control flies, the kinetics of the chaining behavior to playback of the intermittent pulse song was quite similar to that of the continuous pulse song in *rut^2080^* flies (Fig. 6D red and blue lines). When the song pattern was shifted from the continuous pulse song to the intermittent pulse song, however, the decreased chain index of *rut^2080^* flies was restored, as observed in control flies (Figs. 6E and S4C). To compare the chaining behavior between fly strains of different genetic backgrounds, we defined three rates of change: the suppression rate in the first phase of the application of the continuous pulse song, the recovery rate in response to the sound change from the continuous pulse song to the intermittent pulse song, and the drop rate in response to the sound shift from the intermittent pulse song back to the continuous pulse song (Fig. 6E, Table S2, see Materials and Methods for details). Despite the differences in the chain indices immediately after playback onset of the continuous pulse song between wild-type (chain index  = 2.7; Table S1) and *rut^2080^* (chain index  = 1.9; Table S1) flies, their suppression rates were virtually identical (p = 0.16, Student's t-test; Fig. 6F left panel). In contrast, the recovery rates dramatically differed between wild-type and *rut^2080^* flies; recovery was significantly smaller in *rut^2080^* flies than in wild-type flies (Fig. 6F middle panel). This finding indicates that *rut^2080^* flies are less responsive to the shift from the continuous pulse song to the intermittent pulse song. Moreover, as shown in the drop rate, *rut^2080^* flies showed no response when the playback song was changed back to the continuous pulse song (Fig. 6F right panel). This suggests that *rut^2080^* flies had restricted flexibility in the sound-induced response. The unaltered behavior of *rut^2080^* flies during this “drop rate” period was not likely due to a bottom effect of the chaining behavior; the cumulative chain index at the onset of the second playback of the continuous pulse song was significantly higher than that after 20 min of exposure to the second playback (magenta arrows at 40 min and 60 min in Fig. 6E; p<0.01, Wilcoxon signed-rank test; Fig. 6G). Thus, it is unlikely that *rut^2080^* flies retrieved or remembered the formerly experienced playback sound, which was manifested as a suppressed chain index. On the other hand, wild-type flies obviously reproduced the suppression of the chain index observed before the application of the intermittent pulse song (Fig. 6F right panel). These findings strongly suggest that the *rut* adenylyl cyclase is involved in the flexibility and plasticity of sound-induced behavior, at least in male chaining behavior.

## Discussion

Fruit flies and other animals rely on various sensory modalities, e.g., olfactory, gustatory, tactile, auditory, and visual sensory systems, in their courtship behavior [27], [28]. During mating behavior, the nervous system is constantly evaluating incoming stimuli and filtering out those that are not important for making a decision about what to do next. A major stimulus that affects mating behavior in fruit flies is the courtship song, a sound produced by a courting male. When exposed to a courtship song, *D. melanogaster* males in a single-sexed group reportedly show intensive chaining behavior by starting to chase the other flies, possibly because of a dramatic increase in its motivation to find a female [11], [12]. In this study, we developed software that automatically scores the chain index, i.e., the number of flies forming a chain. Using manual methods, scoring the chain index in a single movie file requires approximately 4 h, as opposed to only a few minutes to set up and run the ChaIN system. This capability allows us to systematically compare the effectiveness of various sounds to induce chaining behavior. In contrast to the threshold of the sound to evoke the male-male courtship identified previously (72 dB particle velocity) [10], our measurement was less sensitive with a threshold of 1.1 mm/s particle velocity, which corresponds to 87 dB (Fig. 2B). One possible reason for this discrepancy is the number of males used in a single assay. In contrast to our assay with only 6 males, the previous assay used 24 males for each set [10]. This higher density of males likely increases the probability of male-to-male courtship, as shown in Fig. 2D.

In the present study, we found that sound-evoked chaining behavior in two closely related species, *D. melanogaster* and *D. simulans*, is remarkably robust and is induced preferentially by sound carrying specific IPIs. Electrophysiologic recordings revealed that the neural responses to a pulse song are similar at the level of the auditory sensory neurons in these two species, and are also likely to be similar at the level of the AMMC-A1 neurons, a major secondary auditory neuron type that feeds into the primary auditory center of the fly brain [29]. Taken together, the neurons responsible for the selective IPI preference observed in this study are likely embedded in a brain center higher than the primary auditory center. *D. melanogaster* males exhibited dramatically increased chaining behavior immediately after the sound playback onset, whereas *D. simulans* males exhibited slowly increasing chaining behavior during the sound playback (Fig. 3B, C). Such different kinetics of the chaining behavior are likely due to differences in sex-driven locomotion activity between species; *D. simulans* males reportedly move around less during courtship than *D. melanogaster* males [1]. The lower locomotion activity during the immediate phase of sound playback would thus reduce the chance to find a cognate partner, which would decrease the courtship rate.

Sound-evoked behavioral thresholds have been analyzed using the startle response (1.2×10^−1^ mm/s) or a proboscis extension reflex as an output of sound-associated conditioning (65 dB, which corresponds to 0.9×10^−1^ mm/s) [19], [30]. Precise measurement of the behavioral threshold of the sound-induced chaining behavior is difficult, as both velocity and pressure can vary considerably over small distances in echoic environments such as our behavioral chamber. The maximal velocity that reaches the chamber can be measured at its front end, however, which had a behavioral threshold of 1.1 mm/s particle velocity (87 dB SVL) in our measurement. This is within the level of the natural courtship song (particle velocity ca. 80–95 dB) [31], suggesting that our measurement is within the physiologic range of the fruit fly natural courtship behavior.

Like many other insects, the fruit fly generates acoustic communication signals during both reproductive and agonistic behaviors [1], [20]. Here we showed that the agonistic sound of *D. melanogaster*, which contains longer and more irregular IPIs than its courtship pulse song, evoked chain formation only at the initial phase of the sound playback. Because this agonistic sound seems to have no impact in eliciting a specific behavior in the context of aggression [20], the male-male interactions observed here are likely to represent a courtship chain formation between males. Future studies are required to address the neural mechanism underlying the induction and rapid attenuation of the chain formation with this irregular pulse sound. On the other hand, the chaining response to a recorded song remained high. Moderate irregularities of pulses and dynamic amplitude modulation in the recorded song together might be important for maintaining the behavior.

Sensory adaptation and habituation, the reduction in an animal's response to the repeated occurrence of an unchanging stimulus, is a fundamental process for providing a neural representation of the salience of a stimulus that has recently reached the animal. Adaptation and habituation are common features in all sensory modalities; they are considered to be simple forms of non-associative learning, as well as two of the simplest and most widespread forms of neuronal plasticity [32], [33]. Here, we found that the continuous pulse song leads to a suppression of the sound-evoked chaining behavior of flies, which cannot be explained by motor fatigue or sensory adaptation. Habituation is also improbable, as the behavioral suppression was not released by various dishabituation stimuli. Multiple mechanisms are thus likely involved in this suppression. Moreover, this behavioral suppression depends on the temporal pattern of the pulse burst (Fig. 6A), suggesting that silent intervals between continuous pulses can buffer the suppression. Indeed, male flies exposed to natural courtship songs, which comprise variable sound amplitudes and silent intervals displayed steady chaining behavior when compared with exposure to artificial courtship songs (Figs. 3B and 4B). What is the nature of the behavioral suppression induced by repetitive courtship songs? The behavioral suppression of sound-evoked chaining behavior could be, at least partially, interpreted by a synaptic efficacy change in the central brain. An analysis of excitatory junctional currents in larval neuromuscular junction revealed that the synaptic facilitation elicited by repetitive electric stimuli is much weaker in the *rut* mutant than in controls, even though the initial response to the stimuli is quite normal in the mutant [34]. This is one possible mechanism underlying the partial plasticity in chaining behavior of *rut* flies observed in this study; male *rut* mutant flies displayed weak behavioral plasticity in terms of recovery rate and drop rate (Fig. 6E, F), whereas the chaining behavior seemed to be normal in initial response to the continuous pulse song. These results together suggest the involvement of synaptic regulations exerting a plastic neural response via a cAMP signaling cascade in the suppression of chaining behavior. Additional studies are required to clarify the molecular and neural mechanism underlying this behavioral suppression.

## Materials and Methods

### Animals and Preparation

*Canton-S* flies were used as a wild-type strain for *D. melanogaster*. The *rut* mutant (*rut^2080^*), UAS-GCaMP3, and *D. simulans* flies (stock number: 14021 0251.194) were obtained from the Bloomington Stock Center (Bloomington, IN) and the University of California San Diego *Drosophila* Species Stock Center (La Jolla, CA). A GAL4 driver strain F-Gal4 [35] was used for calcium imaging. Flies were raised on standard *Drosophila* yeast-based media at 25°C at 40 to 60% relative humidity on a 12-h light/dark cycle. For behavioral assays, adult virgin males were collected within 8 h of eclosion; their wings were clipped on the same day following brief induction of anesthesia on ice. Flies between 5 to 7 d after eclosion were used in all experiments.

### Stimulus Design

Artificial songs for the behavioral assay, each of which contains regularly repeated bursts of sine or pulses, were generated using Audacity (The Audacity Team, version 1.3). The intrapulse frequency of the artificial songs was 167 Hz and 333 Hz in the experiments for *D. melanogaster* and *D. simulans*, respectively, which is within the range of pulse songs for each species [18]. A silent period of 2 s was inserted between burst periods of 1 s each, unless otherwise noted. With this stimulus design, the number of pulses in a 1-s sound period differs between IPI series, i.e., the 15-ms IPI song carries 66 pulses in each 1-s sound period, whereas the 105-ms IPI song has 10 pulses. Recorded songs contained regularly repeated 80-s sound clips of either recorded pulse song or agonistic sound (gift from Dr. Ralf Heinrich). In the pulse song with an oscillating IPI, the oscillation period was 58 s with a minimum IPI length of 29 ms and a maximum IPI length of 40 ms. For calcium imaging, continuous pulses with a 35-ms IPI were generated using Spike2 (Cambridge Electronic Design, Cambridge, UK).

### Microphone Recording

An Emkay NR3158 pressure-gradient microphone (Knowles Electronics Inc., Itasca, IL) calibrated with a two-microphone probe (sound intensity probe kit, Type 3599, Brüel & Kjær, Nærum, Denmark) was placed at the front end of the behavior chamber to monitor the particle velocity. Mean amplitudes of the particle velocity in each acoustic stimulus were calculated as the mean peak-to-baseline values of 5 to 10 pulses.

### Behavioral Assay

Behavioral responses of male flies to sound were assayed as described [12]. Briefly, six males were transferred gently to each behavioral chamber (Plexiglas chamber, 50 mm long ×10 mm wide ×6 mm high) and placed in front of a loudspeaker (TAMON, Japan). Synthetic sound signals were delivered from the loudspeaker with an amplifier (RDA-520, Rasteme systems, Japan), placed within 10 cm of the behavior chamber. To observe the contour of the flies clearly, the chamber was backlit from below using an LED light box (ComicMaster Tracer, Too Marker Products, Japan). Video recording, using a monochrome digital camera (Himawari GE60, Library, Tokyo, Japan) equipped with a zoom lens (Lametar 2.8/25 mm, Jenoptik GmbH, Jena, Germany), was started immediately. Flies were not exposed to sound for 5 min, and then exposed to an acoustic stimulus. The recorded movie file, originally 30 frames per second, was down-sampled to 1 frame per second and analyzed offline. To score the chain index, the file was subjected to our newly developed ChaIN method. The chain index in each frame was scored by ChaIN, averaged in every 10 frames, and plotted as a function of time. The probability of chain formation was estimated by dividing a given chain score by the number of flies in the chamber. For a dishabituation test, flies in a chamber were exposed to three types of stimuli: (1) flashes of white light at ca. 1 Hz for 5 s by turning on and off the backlit LED light box manually, (2) 2-s of harsh vortex on a vortex mixer (Taitec, Tokyo, Japan), and (3) 5× bangs of the chamber by picking it up slightly and banging it against the table, during 40-s silent period between two sound periods. To compare the suppression, recovery, and drop rates in the habituation assay, we used the angular transform of the cumulative chain indices for five temporal phases (1-min each, shown in Fig. 6E). Each rate was calculated on the basis of the following equation: (*AC_2_*-*AC_1_*)/*AC_1_*, in which *AC_1_* and *AC_2_* are the angular transforms of the first and second cumulative chain indices, respectively, in a pair of temporal phases. The suppression rate was calculated from the cumulative chain index at the first playback onset of the continuous pulse song (5′ 11″ to 6′10) and at the end of the first playback of the continuous pulse song (23′51″ to 24′50″). The recovery and drop rates were calculated from two cumulative chain indices before and after the sound shift at 25 min (continuous to intermittent) and 40 min (intermittent to continuous; shown by blue vertical lines in Fig. 6E), respectively. All the assays were conducted within 4 h after the start of the light cycle under the condition of 25°C at 40 to 60% relative humidity.

### Statistical Analysis

The statistical analysis was performed using Excel (Microsoft Corporation, Redmond, WA) and its plug-in software Excel-Toukei 2010 (Social Survey Research Information Corporation, Japan). For comparison of more than two groups, the overall difference between groups was determined by Friedman's test or the Kruskal Wallis test. If these tests were significant, differences between individual groups were estimated using Scheffe's test. For comparison of two groups, Mann-Whitney's U test or Wilcoxon signed-rank test was used. In the box plot, upper and lower edges of each box correspond to upper and lower quartiles, respectively. The horizontal line in each box shows the median (50% quartile), and the lines depict the cut-off values. Outliers are indicated by crosses.

### Chain Detection by “ChaIN”

ChaIN can simultaneously analyze four chambers. After subtracting the background image, which was defined as the mean of 40 images randomly picked from the serial grayscale images comprising the movie file, the image series were converted to binary images using Otsu's method [36]. The position of the flies was then computed by fitting an ellipse on the cluster of black pixels (Fig. 1A). Here, the centroid of each binary image was set as a center of the ellipse. The eigenvalues and eigenvector of the covariance matrix in each image were then calculated to give lengths and angles of the major and minor axes. The major axis was then regarded as the body axis of each fly. The head orientation of the flies was computed by applying the Harris & Stephens corner detection algorithm [37] to the silhouette of the flies, because the abdominal tip of a fly is typically sharper than the front part of the head. Chain formation was then defined when this chain area overlapped the body area of another fly (Fig. 1A, B), and the number of flies in the chains was scored as the chain index. The ChaIN software is available at: http://jfly.iam.u-tokyo.ac.jp/chain/.

### Calcium Imaging

To enhance GCaMP3 signals, flies were made homozygous for both GAL4 and UAS-GCaMP3. After raising flies at 29°C for a day to further enhance the GCaMP3 expression, the flies were anesthetized on ice and affixed onto an imaging chamber using silicon grease SH 44M (Toray, Tokyo, Japan) with the ventral side of the fly up. We pulled out the mouthpart of the fly using fine tweezers under a stereomicroscope M125 (Leica Microsystems GmbH, Wetzlar, Germany), to open a window through which we could monitor the fluorescence in the brain. A drop of *Drosophila* Ringer's solution (130 mM NaCl, 5 mM KCl, 2 mM CaCl_2_, 2 mM MgCl_2_, 4 mM, 36 mM sucrose, and 5 mM HEPES [pH 7.3]) [38] was immediately added to prevent dehydration. After removing the small trachea and excessive fats with fine tweezers, the fly, together with the imaging chamber, was fixed on the stage of a fluorescent microscope Axio Imager.A2 (Carl Zeiss, Oberkochen, Germany) equipped with a water-immersion ×20 objective (N.A.  = 0.5) and a spinning disc confocal head CSU-10 (Yokogawa, Tokyo, Japan). This system was equipped with a krypton/argon laser for excitation at 488 nm, a dichroic beam-splitter 405/488/561/640 Di01-T405/488/568/647–13×15×0.5 (Semrock, Rochester, NY), and a band-path filter FF01-528/38–25 (Semrock). The fluorescent image was captured with an EM-CCD camera ImagEM512 (Hamamatsu Photonics, Shizuoka, Japan) at a rate of 3 Hz with an exposure time of 300 ms. The antennal receiver was actuated electrostatically as described previously [39]. Here, the fly was charged to +15 V against ground via a charging electrode inserted into the thorax. We fed voltage commands (from −14 V to +14 V pulses) to a stimulus electrode placed in front of the sound receiver. Time-lapse recordings of GCaMP3 fluorescence were performed by sending trigger signals to the EM-CCD camera. Fluorescent signals between 0 and 1, 10 and 11, and 20 and 21 min after the stimulus onset were imaged and analyzed. The shutter between the laser and the specimen was opened manually only during recordings to prevent bleaching of GCaMP3 fluorescence. Each experiment was performed in at least four flies. The data were analyzed off-line with ImageJ (National Institutes of Health) and Excel (Microsoft Corporation) software. Regions of interest used for analysis were set on the zone A in the AMMC. The intensities of GCaMP3 fluorescence were normalized to those preceding the stimulus onset (t = −2 s). Pseudocolor images of ΔF/F intensity maps were generated using ImageJ.

## Supporting Information

Figure S1 **Chaining behaviors in** ***D. melanogaster*** **and** ***D. simulans*** **flies in response to artificial pulse songs.** Cumulative chain indices of *D. melanogaster* (A) and *D. simulans* (B) flies during three temporal phases are shown. Silent (Top panels), immediate (Middle panels), and later (Bottom panels) phases, indicated in Figs 3B and 3C, are shown. Different letters indicate significant differences between groups (Kruskal-Wallis test followed by Scheffe's multiple comparison, p<0.05). n. s., not significant.
(TIF)

Figure S2 **Behavioral responses of** ***D. melanogaster*** **and** ***D. simulans*** **males.** (A) Response of *D. melanogaster* males to a 105-ms IPI song. Each pulse burst carries either 10 or 29 pulses. Time windows for three temporal phases are hatched in gray (Left panel). Cumulative chain indices during three temporal phases are shown (Right panel) (Mann-Whitney's U test, n. s., not significant). (B) Response of *D. simulans* males to prolonged pulse songs. Sound playback starts at 5 min (dotted vertical line).
(TIF)

Figure S3 **Behavioral suppression to artificial pulse song with various IPIs.** (A) Time-course of the chain index to the continuous pulse song with 35, 55, and 75-ms IPIs. (B) Cumulative chain indices between five temporal phases. Different letters indicate significant differences between groups (Friedman's test followed by Scheffe's multiple comparison, p<0.05). (C) Cumulative chain indices between stimuli of different IPIs. No significant difference was observed between different IPIs at any temporal phase (I to V). n. s., not significant (Kruskal-Wallis test followed by Scheffe's multiple comparison, p>0.05).
(TIF)

Figure S4 **Stimulus-shift induced behavioral changes.** (A–B) A behavioral change in wild-type flies. Sound switch from the intermittent pulse song to the continuous pulse song induced an immediate suppression of the chaining behavior (A). Sound playback starts at 5 min (dotted vertical line). Blue vertical lines depict the time when the sound clip is switched from the intermittent pulse song to the continuous pulse song. Sound shift from the continuous sound of 35-ms IPI to that of 55-ms IPI induced no behavioral recovery (B). Blue vertical lines depict the time when the sound clip is switched. (C) Behavioral changes in *rut^2080^* mutant flies. Shift from the continuous to the intermittent pulse song partially restored the behavioral response (Left panel). When the song pattern was shifted from continuous pulse song (red underline) to intermittent pulse song (blue underline) the decreased chain index was restored (green and pink lines). When the playback song was changed back to the continuous pulse song, however, the chaining behavior was not suppressed (pink line). Blue vertical lines depict the time when the sound clip is switched between two songs. Box plot depicts the comparison of chain indices between five temporal phases (Right panel). Different letters indicate significant differences between groups (Friedman's test followed by Scheffe's test, p<0.05).
(TIF)

Table S1 **Chain indices at each experiment.** C, continuous pulse song; I, intermittent pulse song.
(TIF)

Table S2 **Cumulative chain indices at experiment shown in** **Figure 6E** **.** Numbers in parentheses indicate the angular transform of cumulative chain indices. SR, suppression rate; RR, recovery rate; DR, drop rate.
(TIF)
