# Supplementary figures and images for: Selectivity and Plasticity in a Sound-Evoked Male-Male Interaction in Drosophila
Source: PLoS One. 2013 Sep 24;8(9):e74289. doi: 10.1371/journal.pone.0074289 (PMC3782482; doi:10.1371/journal.pone.0074289)

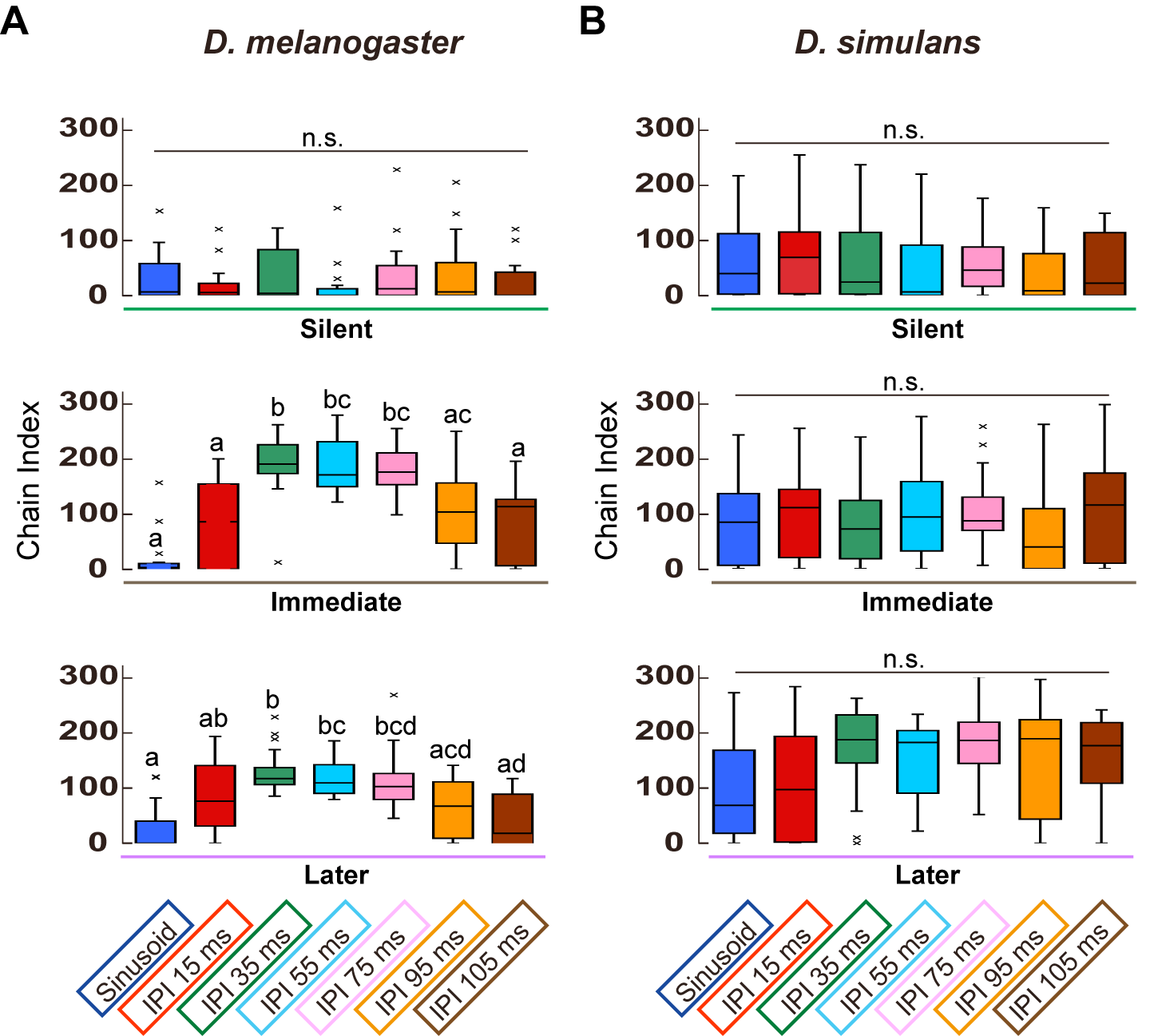

Supplement: Figure S1 — Chaining behaviors in D. melanogaster and D. simulans flies in response to artificial pulse songs. Cumulative chain indices of D. melanogaster (A) and D. simulans (B) flies during three temporal phases are shown. Silent (Top panels), immediate (Middle panels), and later (Bottom panels) phases, indicated in Figs 3B and 3C, are shown. Different letters indicate significant differences between groups (Kruskal-Wallis test followed by Scheffe's multiple comparison, p<0.05). n. s., not significant. (TIF) [file pone.0074289.s001.tif]

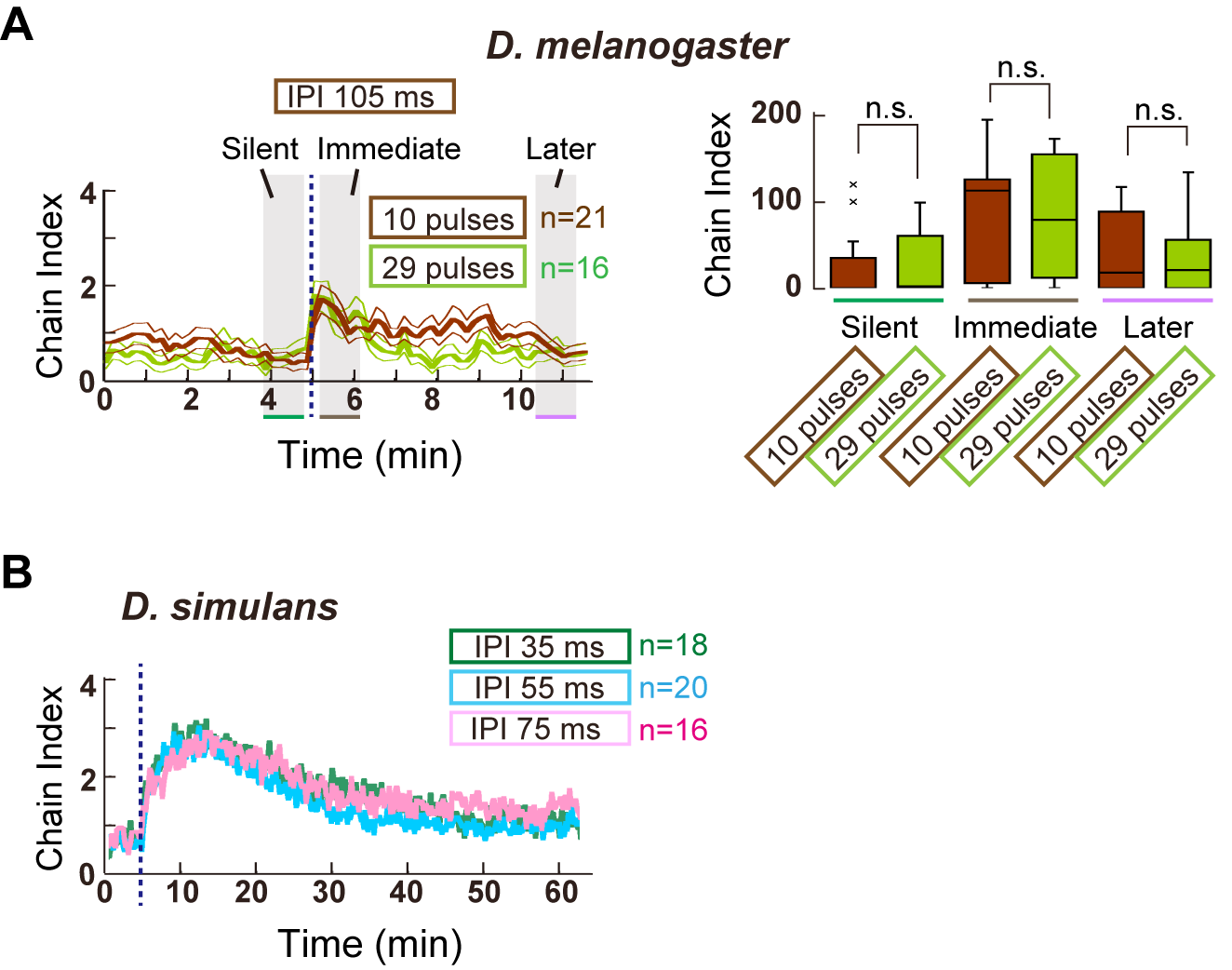

Supplement: Figure S2 — Behavioral responses of D. melanogaster and D. simulans males. (A) Response of D. melanogaster males to a 105-ms IPI song. Each pulse burst carries either 10 or 29 pulses. Time windows for three temporal phases are hatched in gray (Left panel). Cumulative chain indices during three temporal phases are shown (Right panel) (Mann-Whitney's U test, n. s., not significant). (B) Response of D. simulans males to prolonged pulse songs. Sound playback starts at 5 min (dotted vertical line). (TIF) [file pone.0074289.s002.tif]

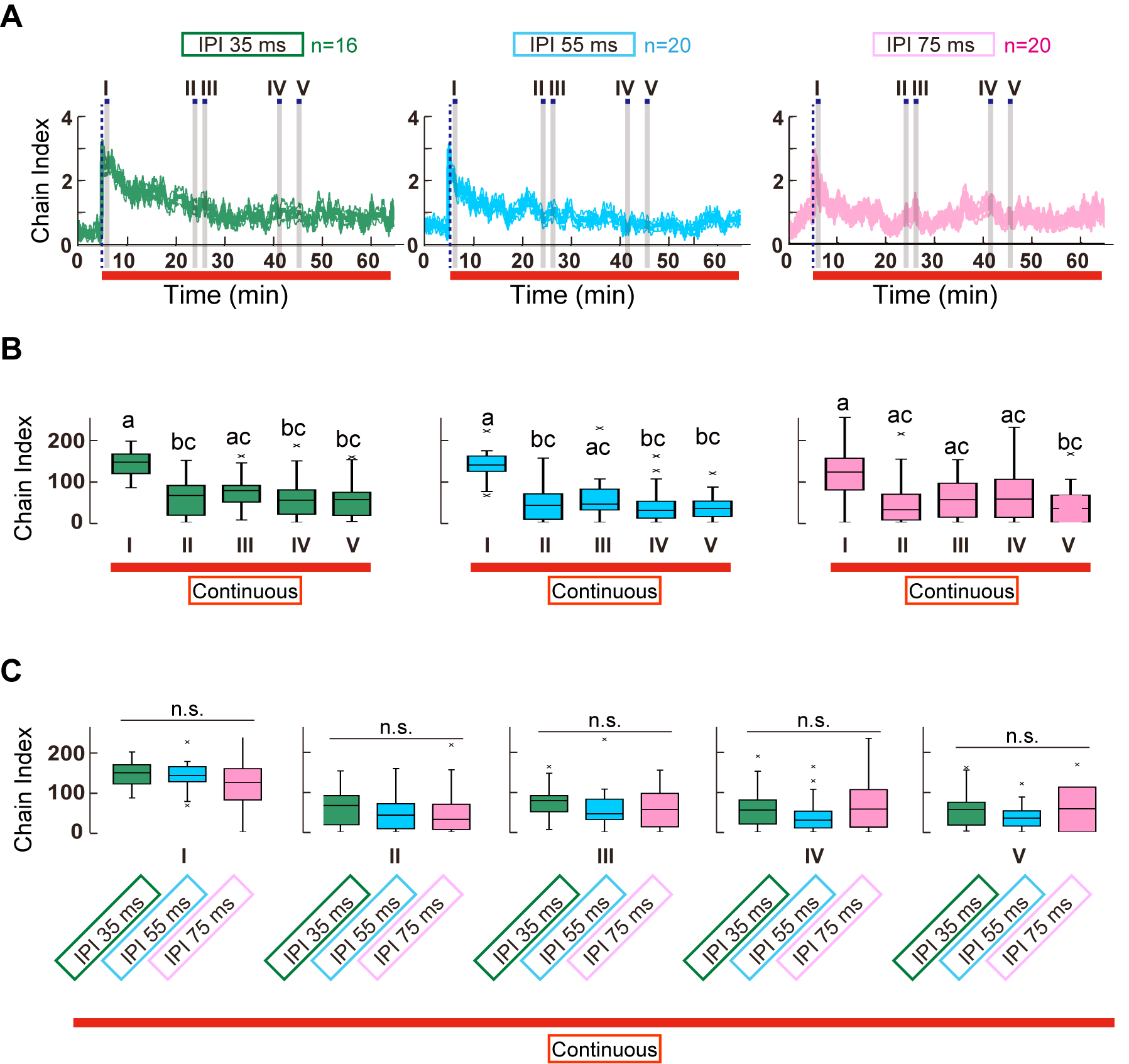

Supplement: Figure S3 — Behavioral suppression to artificial pulse song with various IPIs. (A) Time-course of the chain index to the continuous pulse song with 35, 55, and 75-ms IPIs. (B) Cumulative chain indices between five temporal phases. Different letters indicate significant differences between groups (Friedman's test followed by Scheffe's multiple comparison, p<0.05). (C) Cumulative chain indices between stimuli of different IPIs. No significant difference was observed between different IPIs at any temporal phase (I to V). n. s., not significant (Kruskal-Wallis test followed by Scheffe's multiple comparison, p>0.05). (TIF) [file pone.0074289.s003.tif]

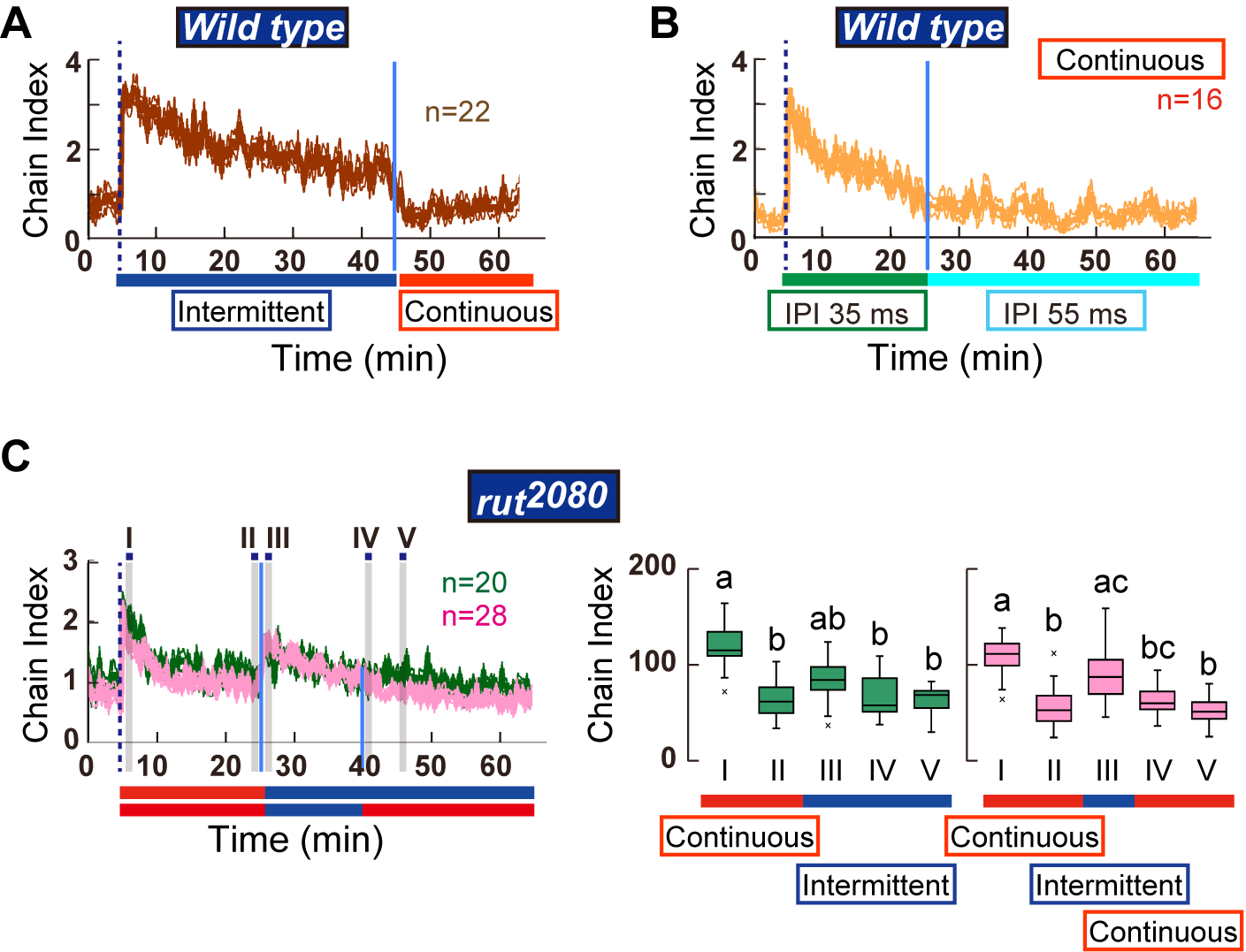

Supplement: Figure S4 — Stimulus-shift induced behavioral changes. (A–B) A behavioral change in wild-type flies. Sound switch from the intermittent pulse song to the continuous pulse song induced an immediate suppression of the chaining behavior (A). Sound playback starts at 5 min (dotted vertical line). Blue vertical lines depict the time when the sound clip is switched from the intermittent pulse song to the continuous pulse song. Sound shift from the continuous sound of 35-ms IPI to that of 55-ms IPI induced no behavioral recovery (B). Blue vertical lines depict the time when the sound clip is switched. (C) Behavioral changes in rut2080 mutant flies. Shift from the continuous to the intermittent pulse song partially restored the behavioral response (Left panel). When the song pattern was shifted from continuous pulse song (red underline) to intermittent pulse song (blue underline) the decreased chain index was restored (green and pink lines). When the playback song was changed back to the continuous pulse song, however, the chaining behavior was not suppressed (pink line). Blue vertical lines depict the time when the sound clip is switched between two songs. Box plot depicts the comparison of chain indices between five temporal phases (Right panel). Different letters indicate significant differences between groups (Friedman's test followed by Scheffe's test, p<0.05). (TIF) [file pone.0074289.s004.tif]

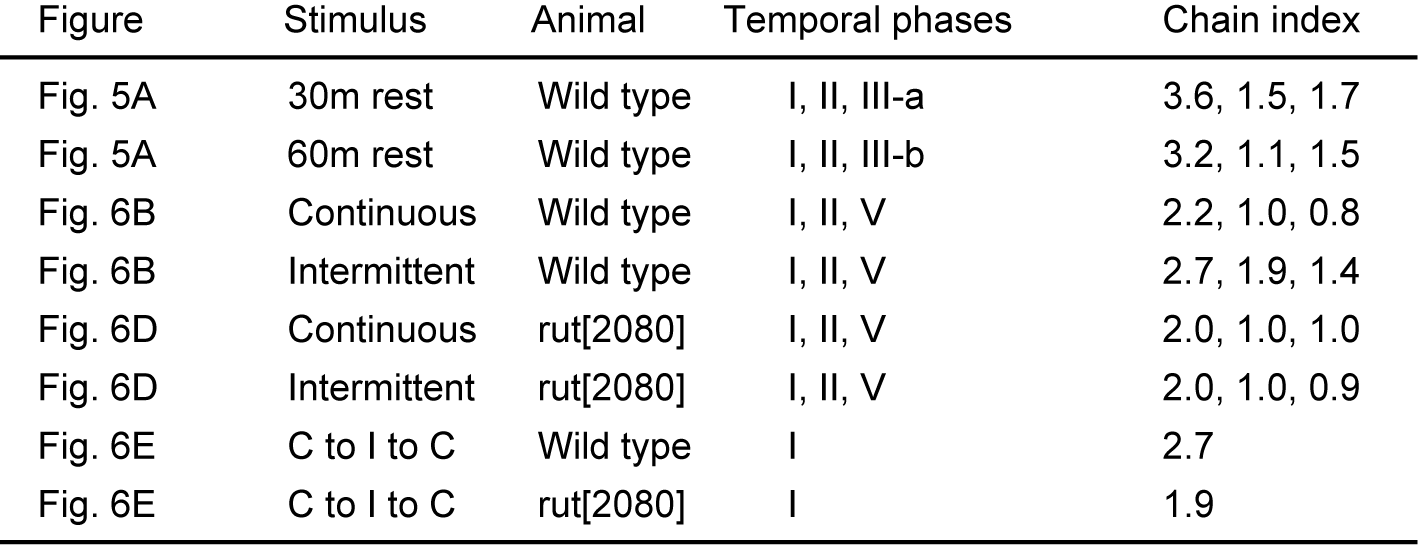

Supplement: Table S1 — Chain indices at each experiment. C, continuous pulse song; I, intermittent pulse song. (TIF) [file pone.0074289.s005.tif]

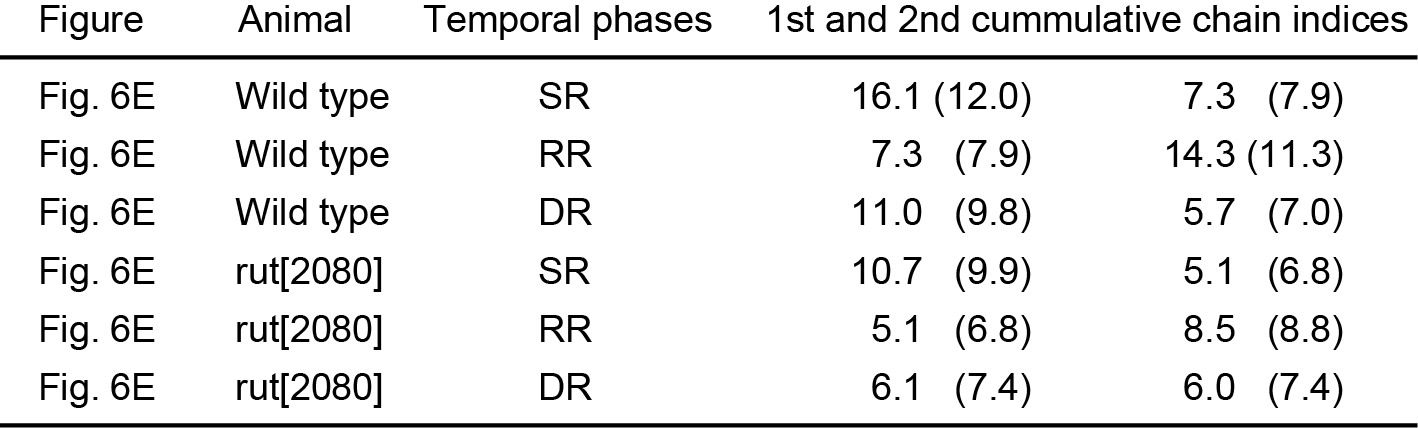

Supplement: Table S2 — Cumulative chain indices at experiment shown in Figure 6E . Numbers in parentheses indicate the angular transform of cumulative chain indices. SR, suppression rate; RR, recovery rate; DR, drop rate. (TIF) [file pone.0074289.s006.tif]
